# cGAS-STING signaling in cardiovascular diseases

**DOI:** 10.3389/fimmu.2024.1402817

**Published:** 2024-05-13

**Authors:** Qianxin Zhang, Lijuan Shen, Hongbiao Ruan, Zhouqing Huang

**Affiliations:** ^1^ Department of Cardiology, The People’s Hospital of Yuhuan, Taizhou, Zhejiang, China; ^2^ Department of Cardiology, The First Affiliated Hospital of Wenzhou Medical University, Wenzhou, Zhejiang, China; ^3^ The Key Laboratory of Cardiovascular Disease of Wenzhou, Department of Cardiology, The First Affiliated Hospital of Wenzhou Medical University, Wenzhou, Zhejiang, China

**Keywords:** CGAS, STING, inflammation, cardiovascular diseases, IFN

## Abstract

Sterile inflammation, characterized by a persistent chronic inflammatory state, significantly contributes to the progression of various diseases such as autoimmune, metabolic, neurodegenerative, and cardiovascular disorders. Recent evidence has increasingly highlighted the intricate connection between inflammatory responses and cardiovascular diseases, underscoring the pivotal role of the Stimulator of Interferon Genes (STING). STING is crucial for the secretion of type I interferon (IFN) and proinflammatory cytokines in response to cytosolic nucleic acids, playing a vital role in the innate immune system. Specifically, research has underscored the STING pathway involvement in unregulated inflammations, where its aberrant activation leads to a surge in inflammatory events, enhanced IFN I responses, and cell death. The primary pathway triggering STING activation is the cyclic GMP-AMP synthase (cGAS) pathway. This review delves into recent findings on STING and the cGAS-STING pathways, focusing on their regulatory mechanisms and impact on cardiovascular diseases. It also discusses the latest advancements in identifying antagonists targeting cGAS and STING, and concludes by assessing the potential of cGAS or STING inhibitors as treatments for cardiovascular diseases.

## Introduction

Cardiovascular diseases (CVDs) represent a wide array of complex conditions, including coronary artery diseases, thoracic aortic aneurysms, heart failure (HF), cardiomyopathies, and congenital cardiac anomalies (1). Their global prevalence highlights a significant impact on health worldwide, contributing to a substantial number of fatalities (2). In 2015, CVDs affected 11 million people in European Society of Cardiology member countries, with a total prevalence of approximately 83.5 million (3). These conditions lead to over 17 million deaths globally each year, including 4 million in Europe alone (4, 5). The interaction between immune responses and inflammation in CVDs’ pathophysiology has become a focal point of recent research. Despite advances in surgery and pharmacology improving clinical outcomes, the overall prognosis for CVD patients remains concerning (6). Immune responses and inflammation are pivotal in the pathogenesis of CVDs, as demonstrated by both innate and adaptive immune responses playing critical roles in atherosclerosis progression. A landmark clinical trial in 2017 showed that targeting inflammation could significantly reduce cardiovascular events in high-risk patients (7, 8). Under pathological conditions, cytoplasmic DNA accumulates through various mechanisms, including efflux from mitochondrial and nuclear DNA into the cytosol and release from dying cells (9, 10). This accumulation triggers the cGAS pathway upon binding with double-stranded DNA, leading to STING activation in the endoplasmic reticulum (11, 12). Activated STING initiates a cascade of immune responses, including the activation of interferon regulatory factor 3 (IRF3), TANK binding kinase 1 (TBK1), and nuclear factor-κB (NF-κB) (13–15). While this process upregulates interferons and inflammatory factors, excessive inflammation can lead to tissue damage and organ dysfunction, exacerbating various diseases (16–18). Inflammation is a key determinant in the onset, progression, and outcomes of CVDs, particularly in atherosclerosis and heart failure, where it significantly contributes to disease progression and adverse outcomes (5, 19). The cGAS-STING signaling pathway has been identified as critical in CVD pathogenesis with recent evidence suggesting that targeting cGAS-STING-mediated inflammation holds promise as a treatment for CVDs (20–22). This comprehensive review explores the impact of cGAS-STING-mediated sterile inflammation on CVDs, including the pathway’s intricate functions and its role in coordinating the IFN immune response. We also examine the landscape of suppressors targeting both cGAS and STING, highlighting potential therapeutic strategies to modulate this crucial pathway.

## Overview of cGAS–STING pathway

**Figure 1:**
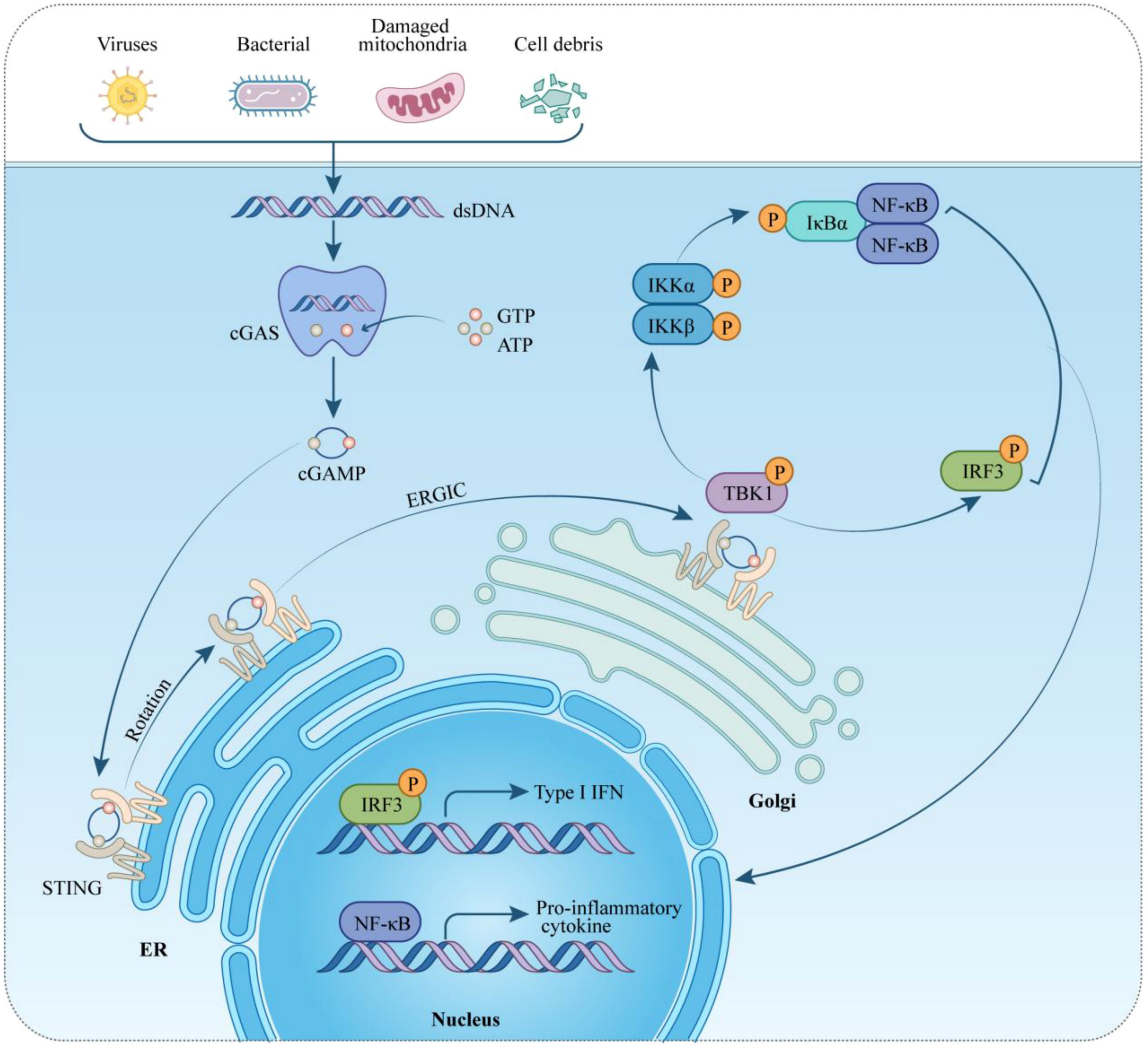
Overview of the cGAS-STING pathway in cardiovascular diseases. Initially, the cGAS-STING signaling pathway serves to protect host cells from pathogens. Further studies have highlighted its significance in the context of cardiovascular diseases, where cGAS detects DNA from both intracellular and extracellular origins. Upon DNA detection, cGAS converts its substrates, GTP and ATP, into cGAMP. STING, situated on the endoplasmic reticulum (ER), detects cGAMP and, when bound to it, becomes activated. This activation leads STING to recruit and phosphorylate TBK1 and IKKs. These kinases then phosphorylate IRF3 and IκBα, respectively. Phosphorylated IRF3 moves to the nucleus, initiating the transcription of type I interferon (IFN-I) genes. Concurrently, phosphorylated IκBα facilitates the recruitment of NF-κB to the nucleus, triggering the transcription of genes encoding proinflammatory cytokines. STING, stimulator of interferon genes; ER, endoplasmic reticulum; ERGIC, ER-Golgi intermediate compartment; TBK1, TANK-binding kinase 1; IRF-3, interferon regulatory factor-3; IκBα: inhibitors of transcription factor NF-κB; type-I IFN, type-I interferon; IL-6, interleukin 6; TNF-α, tumor necrosis factor-α; GTP, guanosine triphosphate; ATP, adenosine triphosphate.

DNA, recognized as a crucial damage-associated molecular pattern (DAMP), triggers the innate immune system by engaging corresponding receptors and initiating intracellular pathways (23). The sources of dsDNA include dying cells, DNA viruses, genomic instability, bacteria, damaged mitochondria, retroviruses, and DNA damage (24, 25). Various DNA sensors, such as absent in melanoma 2 (AIM2), Toll-like receptor (TLR) 9, and cyclic GMP-AMP synthase (cGAS), play pivotal roles in mediating DNA-stimulated innate immune responses (26–28). Among pattern recognition receptors (PRRs), the TLR family, particularly TLR9, is notable (29). Located in the endosomal membrane, TLR9 is highly sensitive to hypomethylated cytidine-phosphate-guanosine regions in DNA from bacteria or viruses, activating the innate immune response (30). Discovered in 2009, AIM2 acts as a cytoplasmic DNA receptor, forming an interaction with dsDNA and associating with the adaptor molecule apoptosis-associated speck-like protein, which activates and recruit caspases (31). This interaction leads to inflammasome/pyroptosome formation, activating both NF-κB and caspase-1 (32). In 2013, Chen et al. identified cGAS, a nucleotides-transferase, that catalyzes the production of the second messenger cGAMP, which binds to STING on the endoplasmic reticulum (ER) membrane, triggering the IFN-I pathway (28). cGAS recognizes both exogenous DNA from pathogens and endogenous DNA from various subcellular locations, such as cytoplasmic chromatin, micronuclei, and mitochondria (33–35). Upon dsDNA recognition, cGAS forms a complex, inducing conformational changes to synthesize cGAMP from ATP and GTP (36, 37). cGAMP binds to STING, causing a conformational change that activates the protein. STING then relocates from the ER to the ER-Golgi intermediate compartment and the Golgi apparatus, activating TBK1. TBK1 phosphorylates itself and STING, activating the IRF3 transcription factor. Dimerized IRF3 translocate into the nucleus, initiating IFN-I synthesis (38–40). Additionally, STING can activate IKK kinase, leading to the phosphorylation of NF-κB transcription factor inhibitors. This cascade ultimately stimulates the production of inflammatory cytokines, including TNF and IL-6 (41) (**Figure 1**).

## The function of cGAS-STING pathway on cardiovascular diseases

Inflammation plays a pivotal role in the progression of cardiovascular diseases. Recent advancements in understanding the STING-mediated inflammatory response have made significant contributions to cardiovascular disease research. This focus has sharpened on the accumulation of cytoplasmic DNA and its role in activating the cGAS-STING-mediated inflammatory response within pathological cardiovascular environments. This section offers a comprehensive review of the pathological processes in cardiovascular diseases, highlighting the regulatory role of inflammation driven by the cGAS-STING pathway (**Table 1**).

**Table 1:** The connection between cardiovascular diseases and the cGAS-STING pathway.

Cardiovascular Disorder	*In vitro* or *in vivo* models	Target	Effects	Reference
Heart failure	Cardiac hypertrophy model, rat cardiac fibroblasts	STING	STING deficiency reduced macrophage infiltration, weakened inflammatory responses, and decreased fibrosis, thus improving cardiac hypertrophy and dysfunction	(42)
Heart failure	Mouse transverse aortic constriction model.	cGAS	Inhibiting cGAS boosted early survival, maintained LV function, and lessened pathological remodeling including hypertrophy, fibrosis, and apoptosis, while reducing early inflammation	(43)
Myocardial infarction	Mouse model of myocardial infarction, fibroblasts and cardiomyocytes	STING	H-151 inhibits the cGAS-STING-IRF3 pathway, reduces inflammation, decreases cardiac fibrosis and preserves myocardial function	(21)
Myocardial infarction	Mice myocardial infraction model	STING	STING inhibitor C-178 reduces infarction size and scarring	(44)
Myocarditis	Sepsis-induced cardiomyopathy mice models	STING	STING knockout attenuates cardiac injury and enhances performance by inhibiting inflammation, apoptosis, and pyroptosis	(45)
Atherosclerosis	Western-type diet-induce Apoe^−/−^ mice	STING	Balasubramide derivative 3C inhibits the JAK2-STAT1-STING pathway, reducing inflammatory response and modulating lipid metabolism and deposition, which improves plaque stability.	(46)
Atherosclerosis	Atherosclerotic patients, western-type diet-induce Apoe^−/−^ mice	STING	C-176 reduces inflammatory molecule expression and macrophage infiltration, alleviating atherosclerosis progression in HFD-fed mice	(22)
Aortic aneurysm and dissection	A sporadic AAD mice model	STING	Amlexanox blocks STING’s Ser366 phosphorylation, effectively mitigating sporadic AAD, maintaining aortic structure, improving survival rates, and reducing the incidence and severity of ADD	(47)
Diabetic cardiomyopathy	DCM mice model and neonatal mouse cardiomyocytes	STING	STING knockdown in diabetic mouse hearts alleviates cardiac pyroptosis and inflammation, prevents diabetes-induced hypertrophy, and restores cardiac function	(20)

## Heart failure

Heart failure (HF) manifests as a syndrome characterized by symptoms such as dyspnea, edema, and lower limb weakness, often accompanied by higher pulmonary edema, peripheral edema, and jugular vein strain (48). It results from impaired heart structure or function, significantly contributing to rising global mortality rates (49). There is mounting evidence linking inflammatory pathways’ activations to the progression of left ventricular remodeling and dysfunction in HF patients, suggesting a potential chronic inflammatory aspect to HF. However, the specific function of inflammation in HF remains incompletely understood. In a study investigating non-ischemic pressure-overload induced HF (transverse aortic constriction, TAC), characterized by cardiac dysfunction, hypertrophy, and fibrosis, elevated IFNα, IFNβ, and STING expressions were observed (42). Remarkably, STING knockout (STING-KO) mice exhibited a return to baseline levels of these indicators, indicating a direct association (42). Similarly, angiotensin II-treated neonatal rat cardiomyocytes displayed heightened STING, IFN-α/-β expressions. Inhibiting STING via siRNA in these cells resulted in a significant reduction in TNFα, IFNα/β, and IL-6/-1β levels. Elevated IFNα/β and STING expressions were also noted in dilative and hypertrophic cardiomyopathies specimens from humans (42). Complementary findings emerged from another study reporting substantial increases in IFN, STING, cGAS, as well as IFN-induced chemokines ISG15, IFIT3, CXCL10 expressions, three days post TAC (43). cGAS silence using adeno-associated virus 9 (AAV9) led to a notable drop in left ventricular remodeling and fibrosis, underscoring the potential therapeutic relevance of the cGAS-STING pathway in HF (43). While scientific investigations have initiated exploration into the relationship between HF and the cGAS-STING signaling pathway, further research is imperative to gain a comprehensive understanding of its precise role in disease progression.

## Myocardial infarction

Myocardial infarction (MI), a severe manifestation of coronary heart disease, significantly impacts patient survival, with the potential for ventricular remodeling, progressive deterioration in cardiac function, and eventual development of chronic heart failure (HF), resulting in a 10-year survival rate typically below 50% (50). Following cardiac cell injury and death during MI, the release of damage-associated molecular patterns (DAMPs) initiates the innate immune response. In a mouse model of MI, activation of interferon regulatory factor 3 (IRF3), a molecule downstream of STING, was found to amplify the release of interferon type I (IFN-I) and inflammation significantly. Disrupting IRF3 signaling markedly reduced the production of inflammatory markers (CXCL10 and IFNB1) and cell infiltration, improving survival and cardiac function post-MI (51). This suggests that inhibiting IRF3 signaling could provide cardiac protection after MI. Rech et al. found that pharmacologic inhibition of STING reduces infarction size and scarring, restoring ventricular systolic function to near-normal levels three weeks post-reperfusion in MI mice, with a noted decrease in myocardial hypertrophy (44). They assessed the inflammatory response by examining expressions of pro-inflammatory genes, uncovering significant differences in IFI44 and CXCL10 expressions between the inhibition and control groups. Hu et al. further confirmed the protective effect of silencing STING through the development of H-151, a selective STING pathway suppressor, both *in vivo* and *in vitro*. STING suppression mitigated the impact of MI by reducing cardiomyocyte apoptosis and fibrosis in fibroblasts induced by the STING pathway in dsDNA-triggered infiltrated macrophages (21). Additionally, inhibiting the macrophage-derived IFN-I signaling pathway, using antibodies against IFN, STING, or a cGAS inhibitor, led to a notable reduction in infarct size in mice subjected to myocardial ischemia-reperfusion (52). These findings collectively suggest that DAMPs released during MI prompt a STING-mediated inflammatory response, contributing to the pathological process of MI.

## Myocarditis

Myocarditis can arise from a variety of sources, including fungi, parasites, viruses, bacteria, autoimmune reactions, or as side effects of drugs (53). Viral infections play a crucial role in the initiation and progression of myocarditis, leading to inflammation that can cause cardiac cells to undergo necrosis or apoptosis, which are key aspects of the disease’s pathophysiological process (54, 55). Recent research has shown that the cGAS-STING pathway has a dual role in viral myocarditis. Following a viral infection, the presence of viral DNA within the cytoplasm can lead to two contrasting outcomes: on one hand, it activates the cGAS-STING pathway, promoting the production of IFN-I, which helps the body combat viruses; on the other hand, this same pathway can exacerbate myocardial damage by inducing inflammation (56, 57). In addition to its role in viral myocarditis, the cGAS-STING pathway is also significant in non-infectious myocarditis and microbial infections. An experimental study using a lipopolysaccharide-induced myocardial infarction mouse model with sepsis observed a notable increase in STING and p-IRF3 expression. Remarkably, STING knockout (STING-KO) effectively reduced inflammation, apoptosis, and cardiomyocyte scarring induced by lipopolysaccharide, while simultaneously improving cardiac function and extending mice survival (45). Myocarditis following Trypanosoma cruzi infection, which activates the poly-ADP ribose polymerase 1-cGAS-NF-κB pathway, facilitating the conversion of inflammatory macrophages, has also been observed. In addition, blocking cGAS activation has proven effective in mitigating this inflammatory response (58).

## Atherosclerosis

Atherosclerosis (AS) is recognized as a chronic inflammatory condition and a primary cause of clinical cardiovascular events. The presence of DNA damage and extracellular vesicles containing DNA at vascular sites contributes to vascular disorders, especially atherosclerosis, by promoting the progression from calcification to plaque formation, and subsequently thrombosis (59). DNA damage is a notable feature in various vascular diseases, including genetic vascular degenerative syndromes (59). While the presence of microbial DNA in human atherosclerotic plaques has been noted, the exact pathophysiological connection to vascular disease progression is yet to be fully understood (60, 61). However, a correlation between the amount of bacterial DNA at plaque sites and the recruitment and accumulation of monocytes suggests a critical role for DNA-modulated monocyte recruitment in both vascular and systemic inflammation (62). Oxidized mitochondrial DNA from macrophages within atherosclerotic plaques is known to induce STING-dependent inflammation, exacerbating atherosclerosis (22). Pham PT et al. reported an accumulation of cytoplasmic DNA and increased cGAMP levels due to DNA damage in the atherosclerotic plaques of mice on a high-fat diet (HFD) (22). Further research indicated that activation of the cGAS-STING pathway in macrophages leads to persistent vascular inflammation and the induction of multiple inflammatory factors (22). Mechanistically, this process may be initiated by the release of mitochondrial DNA induced by the transactive response DNA-binding protein-43 kDa (TDP43) (63). Importantly, either genetic deletion of STING in macrophages or pharmacological blockade of STING has shown to reduce the expression of inflammatory molecules and macrophage infiltration, thereby alleviating the progression of atherosclerosis in mice on an HFD (22, 46).

## Aortic aneurysms and dissections

Aortic aneurysm and dissection (AAD) are closely linked cardiovascular conditions that significantly increase morbidity and mortality risks (64). Currently, effective clinically proven medications to halt the progression of aortic degeneration are absent. AAD may manifest sporadically or in conjunction with genetic disorders. Sporadic AAD is characterized by a progressive loss of smooth muscle cells (SMCs), alongside fragmentation and depletion of the extracellular matrix (ECM), culminating in aneurysm development, dissection, and possible rupture (65). Luo et al. found that inducing sporadic AAD in Sting-deficient mice led to reduced aortic enlargement, decreased elastic fiber fragmentation, and a lower incidence of ADD compared to the wild-type group (47). This finding implies that the absence of STING contributes to the preservation of aortic structure and the smooth muscle layer, underscoring STING’s critical role in the progression of sporadic AAD and aortic degeneration. Further analysis through single-cell transcriptomics of the aorta, GO analyses, and *in vitro* experiments highlighted STING’s involvement in several biological processes, such as response to reactive oxygen species (ROS), DNA damage response, inflammatory response, and cell death in aortic smooth muscle (47). ROS-mediated DNA damage initiates the release of DNA fragments, activating the cGAS-STING-TBK1-IRF3 pathway, which in turn promotes the death of aortic SMCs. It was also discovered that tissue monocytes primarily detect and engulf dsDNA from damaged aortic SMCs, activating the STING-IRF3 pathway and increasing the expression of matrix metallopeptidase 9 (MMP-9), an enzyme that plays a role in ECM degradation. Importantly, the use of a pharmacological TBK1 suppressor to block phosphorylation at STING’s Ser366 was effective in mitigating sporadic AAD. This approach helped maintain the aortic structure, improved survival rates, and reduced the incidence and severity of ADD (47).

## Diabetic cardiomyopathy

Diabetes, a chronic condition, can lead to heart failure due to prolonged cardiac pressure overload (66). Recent research has revealed STING’s role in diabetes-related processes, including cholesterol metabolism, liver inflammation, and islet cell damage (67, 68). Inflammation is a key factor in the development of diabetic cardiomyopathy. Yan et al. demonstrated that activation of the cGAS-STING pathway leads to NLRP3 inflammasome-induced pyroptosis, exacerbating diabetic cardiomyopathy (20). In diabetic mice, hyperlipidemia induces the release of DNA from myocardial cells, which activates the cGAS-STING pathway, resulting in pyroptosis and inflammation. This contributes to myocardial hypertrophy and remodeling (20). However, studies have shown that blocking the Sting gene with AAV9 or pharmacologically suppressing STING can significantly reduce diabetic cardiomyopathy and myocardial inflammation (20, 69). These findings highlight the critical role of cytosolic mtDNA-induced cGAS-STING activation in the pathogenesis of obesity-related diabetic cardiomyopathy (DCM) and provide preclinical evidence supporting the targeting of this pathway as a potential therapeutic strategy for DCM.

## Cardiac aging

Inflammation is a fundamental hallmark of the cardiac aging process. Chronic low-grade inflammation, mediated through specific pathways, is a well-recognized precursor to the development of age-related CVDs (70). Central to this process is mitochondrial dysfunction, which plays a key role in the progression of senescence in CVD (71). One critical outcome of mitochondrial dysfunction is the release of mitochondrial DNA (mtDNA) into the cytoplasm (72). This event can activate various inflammatory pathways, including the cGAS pathway. cGAS, stimulated by the presence of cytosolic DNA, serves as a potent activator of the STING pathway. Upon activation, STING induces the transcription factor interferon-regulatory factor 3 (IRF3) to enter the nucleus, promoting the secretion of interferons and further enhancing the inflammatory response (73, 74). Recent research by Wang et al. reported that nanoplastics cause cardiomyocytes senescence by triggering a series of senescence-related marker molecules (75). Further experiments showed that nano-scaled microplastics may lead to ROS production by inducing calcium overload. The excessive accumulation of ROS leads to the release of mtDNA from mitochondria into the cytoplasm, which in turn activates the cGAS-STING signaling pathway, thus leading to cardiomyocyte senescence (75). In light of these findings, targeting the STING pathway may represent a novel therapeutic strategy in managing age-associated cardiovascular conditions by moderating the senescent inflammatory processes that significantly contribute to disease progression.

## Discovery of cGAS-STING-targeting drugs

**Figure 2:**
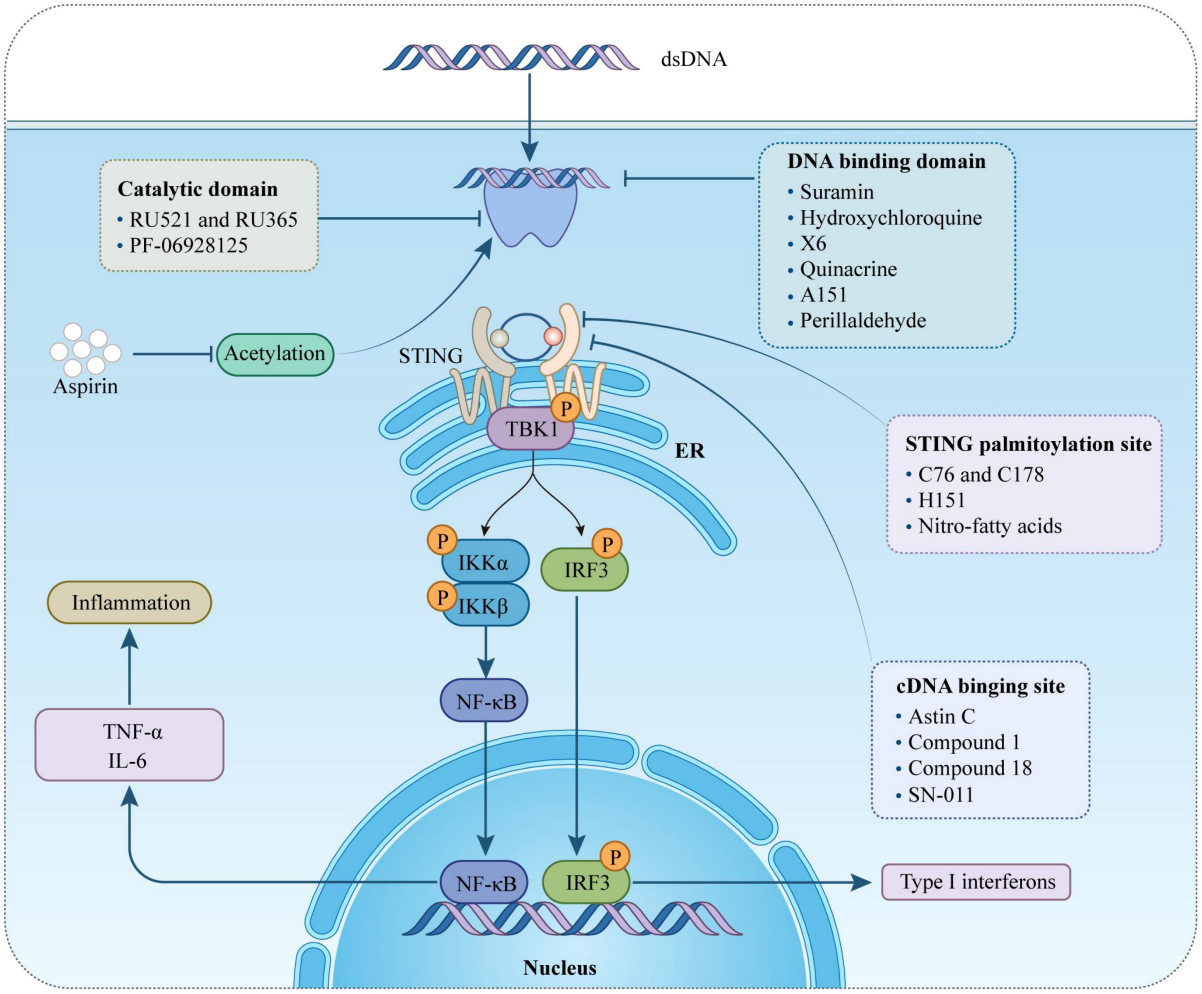
Therapeutical potential of cGAS-STING pathway targeting against inflammation in cardiovascular diseases. This figure provides an overview of different pharmacological agents to modulate the cGAS-STING signaling pathway by intervening at various targets. STING, stimulator of interferon genes; ER, endoplasmic reticulum; TBK1, TANK-binding kinase 1; IRF-3, interferon regulatory factor-3; IκBα, inhibitors of transcription factor NF-κB; type-I IFN, type-I interferon; IL-6, interleukin 6; TNF-α, tumor necrosis factor-α.

As previously emphasized, the cGAS-STING signaling pathway plays a pivotal role in the progression of various sterile cardiovascular diseases (CVDs). This pathway has emerged as a critical therapeutic target not only for the induction of cytokines, including IFN-I, but also in addressing broader health challenges. Several small-molecule agonists targeting this pathway have been developed and are actively being tested in clinical trials (NCT03172936, NCT04144140) (76). These efforts highlight the potential of modulating this pathway. Currently, treatments such as the JAK inhibitor baricitinib and steroids are used clinically to manage patients with STING activation (77, 78). However, it is necessary to develop novel drug interventions that more directly target cGAS and STING. This section will explore the use of cGAS and STING suppressors specifically in the context of cardiovascular diseases, analyzing their potential therapeutic benefits and implications. By targeting these molecular mechanisms, novel treatment strategies aim to offer more precise control over the inflammatory processes that contribute to the progression of CVDs, providing a promising avenue for therapeutic intervention (**Figure 2**) (**Table 2**).

**Table 2:** Overview of cGAS-STING signaling pathway inhibitors.

Target	Inhibitor(s)	Molecular mechanism	Inhibitory potency	Reference
cGAS	Aspirin	Acetylating cGAS at three lysine residues and blocking the activity of cGAS	IC_50 =_ 0.760mM, BMDMs from Trex1^–/–^ mice	(79)
cGAS	A151	Inhibiting the binding of DNA to cGAS	IC_50 =_ 165 nM, THP-1 cells	(80)
cGAS	hydroxychloroquine (HCQ)	Prevents the formation of the cGAS-dsDNA complex by binding to DNA at the cGAS/DNA interface	NA	(81)
cGAS	Suramin	Inhibits cGAS activity by blocking its dsDNA binding domain and displacing dsDNA	NA	(82)
cGAS	Quinacrine (QC)	Inhibits cGAS activation by obstructing its dsDNA binding	IC_50 =_ 3.7 μM, THP-1 cells	(81)
cGAS	X6	Disrupts the binding between dsDNA and cGAS, thereby suppressing cGAS activation	IC_50 =_ 14 μM, THP-1 cells	(83)
cGAS	RU.521	Inhibits the catalytic activity of ATP and GTP sites on cGAS, thereby affecting cGAMP production	IC_50 =_ 700 nM, RAW cells	(84)
cGAS	PF-06928215	Targeting the catalytic site	IC_50 =_ 4.9 μM, cGAS enzymatic assay	(85)
STING	SN-011	Inhibits STING by blocking the CDN binding domain	IC_50 =_ 76 nM, L929 cells; IC_50 =_ 502.8 nM, HFFs	(86)
STING	Astin C	Targeting the CDN-binding domain	Isothermal titration calorimetry K_d_ = 53 nM	(87)
STING	Compounds 18	Inhibits STING by blocking the CDN binding domain	IC_50 =_ 11 μM, THP-1 cells	(88)
STING	Nitro-fatty acids	Targeting the palmitoylation site	NA	(89)
STING	C-178/C-176	Covalently bind to Cys91 residue site of STING	NA	(90)
STING	H-151	Binds to the Cys91 residue on the STING protein, inhibiting its palmitoylation.	IC_50 =_ 100 nM, MEFs and HFFs	(90)
STING	Amlexanox	Blocks TBK1-induced phosphorylation at Ser366, preventing STING activation.	NA	(91)

## cGAS inhibitors

cGAS acts as both a molecular alarm and an enzyme, catalyzing the production of cGAMP for STING signaling. The development of small molecule cGAS suppressors is based on three distinct mechanisms: The first is that mediating cGAS post-translational modification (92); The second is blocking the binding of DNA to cGAS (46); The third is targeting the catalytic pocket of cGAS (59). In the realm of cGAS inhibitors, the first mechanism focuses on the post-translational modifications of cGAS, with Aspirin being an example that targets cGAS acetylation at Lys 384/394/414 to effectively suppress autoimmunity caused by TREX1 genetic deletion (79). The second mechanism is aimed at interrupting the interaction between cGAS and dsDNA. Compounds such as A151, hydroxychloroquine (HCQ), Suramin, quinacrine (QC), and X6 prevent the formation of the cGAS-dsDNA complex by binding to the DNA minor groove at the cGAS/DNA interface (79–81, 83). Suramin, a nucleic acid mimic, competes with dsDNA for the cGAS binding site, reducing the migration and proliferation of vascular smooth muscle cells (VSMCs) and decreasing neointima hyperplasia (82, 93, 94). The third mechanism involves inhibitors that compete with ATP or GTP, the natural substrates of cGAS, or with cGAMP, the product. This category includes non-substrate-competitive suppressors like RU.521/365, which target essential residues in the catalytic site to reduce binding to ATP/GTP and, consequently, downregulate IFN expression. RU.521 specifically binds to the cGAS catalytic sites, inhibiting cGAMP synthesis and subsequent STING activation, and has been shown to protect against septic cardiomyopathy induced by LPS (84, 95). Meanwhile, substrate-competitive inhibitors, such as PF-06928215, directly inhibit the catalytic activity of cGAS and have been proven to alleviate palmitic acid-induced contractile dysfunction in cardiomyocytes (85, 96). Overall, cGAS inhibitors demonstrate protective effects in cardiovascular diseases.

## Inhibitors of STING

STING is a critical signaling molecule in the cGAS–STING DNA-sensing pathway, with its activation essential for various intracellular signaling cascades. Development of STING inhibitors has focused on the palmitoylation site and ligand-binding pocket, utilizing computer-aided design (97, 98). This led to the identification of candidate molecules through high-throughput screening, with their effectiveness confirmed in mouse or human models. Suppressor designs aim to block STING activation by its endogenous ligand, cGAMP, with notable examples including SN-011, the natural cyclic peptide Astin C, and tetrahydroisoquinoline compounds (1 and 18) (86–88). Compound 18, targeting the CDN-binding domain (CBD) of STING, binds to the STING homodimer in an inactive ‘open’ conformation, as shown by crystallographic studies. Despite modest antagonistic effects *in vitro*, it offers favorable oral exposure (88). Astin C, from Aster tataricus, disrupts IRF3 recruitment to the STING signalosome while maintaining the STING-TBK1 interaction (87). Gong et al. highlighted Astin C’s potential to alleviate palmitic acid-induced cardiomyocyte contractile dysfunction by inhibiting the cGAS-STING pathway (96). STING palmitoylation at Cys88/91, crucial for signal transduction, is facilitated by palmitoyltransferases within the Golgi apparatus (15). Nitro-fatty acids accumulate during viral infections and inhibit STING activation through nitroalkylation at its palmitoylation site, showing protective effects against various cardiovascular diseases (89, 99–101). CXA-10, a well-tolerated nitro-fatty acid, is under phase II clinical trials for pulmonary hypertension (NCT04125745/NCT04053543/NCT03449524) (18). Nitrofuran derivatives like C-178, H-151, and C-176, identified via cell-based chemical screening, specifically target Cys91 on STING, blocking activation-induced palmitoylation (90). These compounds have shown efficacy in cardiovascular disease models, displaying anti-inflammatory effects. C-176 and H-151 protect against cardiomyopathy, including myocardial infarction, ischemia-reperfusion injury, and diabetic cardiomyopathy (21, 44, 69). C-176 also suppressed atherosclerosis and chronic kidney disease (CKD) development in HFD-induced Apoe^−/−^ mice (22, 102). Notably, C-176/178 target mouse STING, whereas H-151 is effective against both mouse and human STING. H-151 disrupts STING activation’s necessary palmitoylation and prevents the assembly of the STING polymer complex, reducing infarct dilation and scar formation, thus restoring left ventricular systolic function and reducing myocardial hypertrophy in a myocardial infarction mouse model (44, 90). Additionally, targeting posttranslational modifications of STING and its associated kinases presents viable methods for inhibiting STING activity. For example, the FDA-approved drug amlexanox, known for its anti-inflammatory effects, is utilized in clinical settings to treat asthma and recurrent aphthous ulcers (103). Amlexanox effectively prevents complete activation of STING by blocking TBK1-induced phosphorylation at Ser366, due to its strong affinity and specificity for TBK1 (91). In summary, STING inhibitors have shown protective effects against cardiovascular diseases, presenting a novel strategy for treatment.

## Conclusion and perspective

In the realm of cardiovascular diseases, the persistence of an inflammatory response significantly impacts initiation, development, and outcomes, often leading to adverse clinical consequences (19, 104). The cGAS-STING pathway, recognized for its role in mediating sterile inflammation, has increasingly become a focus of interest over the years (17). Pathological scenarios such as mitochondrial damage or cell death in immune cells, vascular endothelial cells, vascular smooth muscle cells, or cardiomyocytes can cause mitochondrial or nuclear DNA to be released into the cytoplasm (18). This cytoplasmic DNA then activates the cGAS-STING pathway, upregulating inflammatory factors, chemokines, and interferons, thereby exacerbating the inflammatory response (105). This review highlights the critical association between cardiac dysfunction and the cGAS-STING pathway, which is central to cell death and influences various physiological functions. The pathway is instrumental in the onset and progression of diverse cardiovascular disorders, such as MI, HF, and myocarditis, by mediating inflammation, regulating immunity, promoting autophagy, and contributing to aging. Animal studies have consistently shown that inhibiting this pathway can improve cardiac function in these diseases, also leading to increased survival rates. Given these promising findings, the cGAS-STING pathway is considered a potential therapeutic target for various cardiovascular disorders. Nonetheless, due to the complex and varied nature of cardiovascular diseases, further exploration and a deeper understanding of the cGAS-STING pathway’s role in different cardiovascular conditions are essential.

## Author contributions

QZ: Conceptualization, Data curation, Resources, Writing – original draft. LS: Investigation, Methodology, Validation, Writing – review & editing. HR: Supervision, Validation, Visualization, Writing – review & editing. ZH: Conceptualization, Funding acquisition, Resources, Validation, Writing – original draft, Writing – review & editing.
